# Long‐Term Benefits Following Hepatitis C Cure Through Facilitated Telemedicine; Experiences of People With Opioid use Disorder Five Years After Achieving a Sustained Virological Response

**DOI:** 10.1111/hex.70404

**Published:** 2025-09-24

**Authors:** Zoi Papalamprakopoulou, Tin H. Dang, Christopher J. Gonzalez, Colin J. Tedesco, Paul F. Updike, Christina Corl, Arpan Dharia, Noelia Hernandez, Martin F. Shapiro, Elaine Wethington, Andrew H. Talal

**Affiliations:** ^1^ Division of Gastroenterology, Hepatology, and Nutrition, UB‐CTRC, Jacobs School of Medicine and Biomedical Sciences University at Buffalo State University of New York Buffalo New York USA; ^2^ Division of General Internal Medicine, Department of Medicine Weill Cornell Medicine New York New York USA; ^3^ Pathways—Buffalo, Catholic Health Buffalo New York USA; ^4^ Pathways—Rochester, Catholic Health Rochester New York USA; ^5^ Department of Psychology, College of Human Ecology Cornell University Ithaca New York USA; ^6^ Department of Sociology, College of Arts and Sciences Cornell University Ithaca New York USA

**Keywords:** hepatitis C, opioid use disorder, opioid use disorder recovery, sustained viral response, telemedicine

## Abstract

**Background:**

Facilitated telemedicine is highly effective for hepatitis C virus (HCV) treatment among people with opioid use disorder (OUD). However, the long‐term impact of sustained virological response (SVR) through this model of treatment remains unexplored. We examined how people with OUD perceive SVR achieved through facilitated telemedicine.

**Methods:**

We conducted two focus group discussions (FGDs) with nine participants at least 5 years post‐SVR through a randomised controlled trial of facilitated telemedicine in opioid treatment programmes (OTPs) (New York State, 2018–2020). Eligibility required active OTP enrolment at the time of FGDs. We used a semi‐structured interview guide and performed thematic analysis of FGD transcripts.

**Results:**

Participants had a mean age of 52.6 years (SD = 13.7), 66.6% were male, and 88.8% identified as White. We identified three FGD themes, each corresponding to pre‐, during and post‐intervention phases (see Figure 1): (1) Attitudes towards HCV and barriers to treatment among people with OUD, (2) Embracing facilitated telemedicine for HCV care integrated into OTPs, and (3) Experiencing long‐term benefits from facilitated telemedicine across all aspects of HCV care and overall well‐being. Barriers included competing priorities, perceiving HCV treatment as a low priority, and insurance restrictions (Theme 1). Participants valued facilitated telemedicine for its convenience, empathetic clinicians, and one‐stop shop approach (Theme 2). At least 5 years post‐SVR, participants reported benefits in HCV knowledge, improved OUD recovery, and improvement in whole health (Theme 3).

**Conclusion:**

At least 5 years post‐SVR, people with OUD reported improvements in OUD recovery, overall well‐being and sustained satisfaction with telemedicine‐based HCV care. These findings highlight the lasting impact of both an SVR and care delivery through facilitated telemedicine.

**Patient and Public Contribution:**

In this study of patient involvement, we conducted focus groups with patient‐participants to examine the long‐term impact of receiving HCV care through facilitated telemedicine integrated into OTPs. Participants had previously taken part in a randomised controlled trial of facilitated telemedicine (New York State, 2018–2020). At least 5 years after achieving an SVR, we sought participant feedback to evaluate the long‐term impact and sustainability of facilitated telemedicine as an approach to achieve an HCV cure with the objective of informing future policy development. Participants had also contributed critical input at various stages of the original study's design and implementation. During the pilot phase, participants advocated for facilitated telemedicine in a testimonial video. Participants provided feedback on design and implementation by participating in planning and site initiation meetings. A Patient Advisory Committee ensured participant voices were integrated into the research process by representing their feedback on study conduct. Additionally, a Sustainability Committee supported public involvement by promoting educational opportunities, providing input on implementation, and addressing long‐term sustainability considerations.

## 1 Introduction

Chronic hepatitis C virus (HCV) infection, when left untreated, can progress to cirrhosis, liver cancer and ultimately increased mortality [1]. Treatment with direct‐acting antivirals (DAAs) enables viral clearance in over 95% of cases within 8–12 weeks [2]. These regimens are simple, well‐tolerated and highly effective. Despite the availability of highly curative treatment, people with opioid use disorder (OUD), a population at the highest risk for HCV and a priority for HCV elimination strategies, face complex social and medical barriers to accessing HCV care [3]. These challenges include low socio‐economic status, financial hardship, homelessness, the often asymptomatic nature of HCV resulting in delayed care‐seeking, difficulties navigating the healthcare system, limited health literacy, and pervasive stigma and discrimination within conventional healthcare settings [4, 5]. Furthermore, the standard‐of‐care pathway for HCV treatment, which typically involves referral to a hepatitis specialist, has shown limited effectiveness for this population. Data indicate high rates of loss to follow‐up in the referral stage, with studies reporting treatment initiation rates of less than 20% in community‐based settings, suggesting even lower rates when referral occurs outside of community organisations [6]. Consequently, achieving the U.S. National HCV Elimination goals, which require that 80% of people with HCV achieve sustained virologic response (SVR) by 2030, remains unlikely without reshaping healthcare delivery models [7].

To bridge gaps in HCV care for people with OUD, healthcare systems should adopt innovative, decentralised, patient‐centred models that integrate medical care, harm reduction, OUD treatment and social services. These models aim to meet individuals where they are, often outside traditional healthcare settings [8, 9]. One such promising approach is the facilitated telemedicine model, which leverages technology to expand access to HCV care for people with OUD [10, 11, 12]. This model involves real‐time, two‐way videoconferencing between the patient and a remote physician, supported by an onsite facilitator [13]. The telemedicine facilitator assists with scheduling virtual appointments, operating digital equipment, communicating with the healthcare providers during virtual visits, coordinating HCV medication delivery, and ensuring treatment adherence. Facilitators may be peers with lived experience of substance use or trained staff members who may also serve as case managers, in some settings. The term case manager refers to a broader role that extends beyond facilitating telemedicine appointments to coordinating comprehensive medical and behavioural patient care. Lukersmith et al. outlined the responsibilities of case managers to include case finding, establishing rapport, assessment, planning, navigation, provision of care, implementation, coordination, monitoring, evaluation, feedback, patient education, advocacy, supportive counselling, administration, discharge, and community service development [14]. In research settings, case managers may also take on study‐related responsibilities, such as participant recruitment and data collection [12]. Facilitated telemedicine is typically integrated within opioid treatment programmes (OTPs), syringe service programmes (SSPs) and other community‐based sites where people with OUD already receive services [8, 15]. This integration not only improves access to HCV care but also promotes virtual healthcare equity by addressing the digital divide that often prevents marginalised populations from benefiting from virtually delivered healthcare [16].

Recent research supports the effectiveness of facilitated telemedicine for HCV care [17, 18]. A randomised trial by Seaman et al. in rural Oregon showed that 85% of telemedicine participants initiated treatment (vs. 12% in enhanced usual care) and 63% achieved SVR at 12 weeks (vs. 16% in enhanced usual care) [19]. Another multicentre study conducted in Spain reported that 86% of participants initiated treatment through telemedicine, 84% completed treatment and 93% achieved an HCV cure [20]. In our previous investigation, we conducted a randomised controlled trial of facilitated telemedicine integrated in OTPs throughout New York State. In facilitated telemedicine, 92% of participants initiated treatment and 90% achieved SVR, compared to 40% and 39%, respectively, in usual care [15]. Substance use also decreased significantly among cured participants with minimal reinfections during 2 years of follow‐up. In addition, we documented high levels of patient satisfaction 6 months after study completion [21]. Research consistently shows that patient satisfaction is a key factor in promoting sustained engagement in care, which in turn improves long‐term health outcomes [22]. However, despite these encouraging short‐term results, insights into the long‐term impact of achieving SVR through facilitated telemedicine, particularly from the patients' perspectives, are largely absent from the literature. For an intervention to meaningfully inform public health policy and contribute to national HCV elimination efforts, its sustainability and long‐term effectiveness must be thoroughly evaluated [23].

We aimed to explore the long‐term perceptions of people with OUD on an HCV cure achieved through facilitated telemedicine at least 5 years after completing treatment. By assessing these views, we seek to generate actionable insights to inform strategies that promote sustainable and equitable HCV elimination efforts.

## 2 Materials and Methods

We conducted two focus group discussions (FGDs) with nine people with OUD to examine their long‐term perspectives on an HCV cure obtained through facilitated telemedicine. Our study participants had previously received HCV treatment with DAAs through facilitated telemedicine between 2018 and 2020 as part of a multisite pragmatic trial conducted in 12 OTPs across New York State. Details on the study design are available elsewhere (ClinicalTrials.gov NCT02933970) [15, 24]. The study adhered to the Helsinki Declaration principles. We obtained written informed consent from all study‐eligible participants before their participation and verbal consent before recording the FGDs.

### 2.1 Study Population and Recruitment

We employed purposive sampling to recruit participants from two OTPs in New York State. Inclusion criteria included individuals aged at least 18 years who had previously achieved SVR through facilitated telemedicine, remained active patients at the OTPs where they had received HCV treatment, and were able to provide written informed consent. To facilitate recruitment, we employed study facilitators (C.C. and C.J.T.) who were employed by the OTPs and are certified physician assistants. Initially, we identified a list of potential participants using a trial‐specific database from the original trial period. Study facilitators then confirmed the current patient status by cross‐referencing this list with OTP electronic health records. Through a warm hand‐off process mediated by the facilitators, a research team member (Z.P.) met with potential participants to assess their willingness to participate and confirm their eligibility. She explained all aspects of study involvement, obtained written informed consent, and led the FGDs.

### 2.2 Data Collection

We conducted two FGDs in October 2024 and January 2025, with 4 and 5 participants, respectively (*n* = 9). The FGDs were led by an interviewer experienced in qualitative research (Z.P.) [5, 25]. Each FGD lasted between 60 and 90 min and was held in a private room at the OTP. We did not expect that participation in the FGDs would result in significant distress or harm. At the initiation of the FGDs, the research team emphasised that participation was entirely voluntary and that participants were free to terminate their FGD participation at any time without explanation or penalty. To protect participant identities, we encouraged the use of pseudonyms during the FGDs. Participants were reimbursed with a $50 Visa gift card for their time and effort. No participants prematurely discontinued the FGDs.

We developed a semi‐structured interview guide featuring open‐ended, non‐stigmatising and non‐leading questions to facilitate discussions about prior experiences with HCV treatment and the facilitated telemedicine intervention. Example questions included: ‘*What did you know about hepatitis C before getting tested in the prior research study?’*, ‘*What do you think about having received hepatitis C treatment through telemedicine?’* and ‘*How has your life changed since being cured of hepatitis C?’* The complete interview guide is included in Additional File 1. To encourage more in‐depth discussions, we used probes to further explore participants' perspectives and experiences. An expert in qualitative research design (E.W.) ensured the appropriateness of the interview guide for the target population, with input from all study team members.

The FGDs were audio‐recorded and professionally transcribed by the qualified external language service provider Ubiqus/Acolad. The transcripts were de‐identified before being shared with the research team. During the de‐identification process, participants' real names and any pseudonyms used in the FGDs were replaced with labels indicating the FGD session and participants' seating order. For example, a participant labelled ‘F1P2’ attended the first FGD and was assigned the number 2 based on their seating arrangement. At the conclusion of each FGD, we obtained participants' demographic information through a brief questionnaire, including self‐reported age, sex, ethnicity, race and educational level. Interview recordings, transcripts, and demographic data were stored in an encrypted password‐protected server.

### 2.3 Analysis

We analysed the FGD transcripts using thematic analysis. The analysis team was comprised of four research members, referred to as ‘analysts’ (Z.P., T.H.D., C.J.G. and A.H.T.), under the guidance of an expert in qualitative methodology (E.W.). The analysis followed an iterative process, beginning with independent analysis and coding of the FGD transcripts by each analyst following the completion of all FGDs. At this stage, analysts coded the transcripts, identified sub‐themes, consolidated the themes into preliminary higher‐level themes, and selected representative quotations. The team then held a series of weekly meetings to discuss the preliminary findings, refine themes, and resolve disagreements. When disagreements arose, analysts revisited the original recordings and transcripts until a consensus was reached. In the final stage, all team members provided input on the findings. At the conclusion of the process, all study team members agreed on the findings and produced a comprehensive codebook that is included in Additional File 2. We determined that thematic saturation was reached when no new themes or insights emerged, even with the potential inclusion of additional FGDs. Our study adheres to the Consolidated Criteria for Reporting Qualitative Research (COREQ) [included in Additional File 3].

## 3 Results

**Figure 1:**
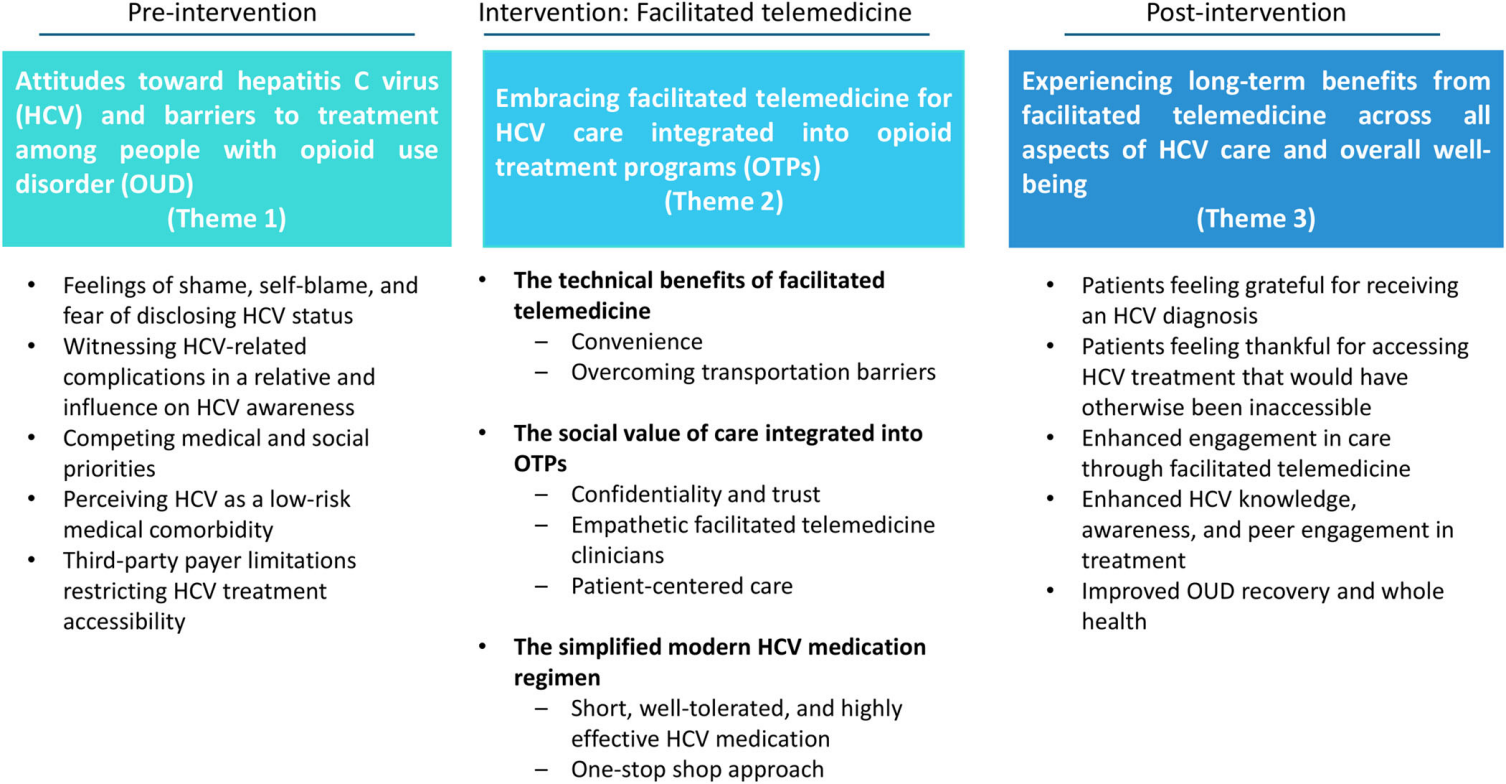
HCV care barriers included competing medical and social priorities, perceiving HCV treatment as of low priority, and third‐party payer restrictions (Theme 1). Participants valued facilitated telemedicine for its convenience, empathetic clinicians, and one‐stop shop approach (Theme 2). At least 5 years post‐SVR, participants reported benefits in HCV knowledge, improved OUD recovery, and improvement in whole health (Theme 3).

Participants had a mean age of 52.6 years (standard deviation = 13.7), 66.6% (6/9) were male, and 88.8% (8/9) identified as White. We identified three FGD themes each corresponding to pre‐, during or post‐intervention of facilitated telemedicine: (1) attitudes towards HCV and barriers to treatment among people with OUD, (2) embracing facilitated telemedicine for HCV care integrated into OTPs, and (3) experiencing long‐term benefits from facilitated telemedicine across all aspects of HCV care and overall well‐being (Figure 1). The table illustrates the key FGD themes, sub‐themes, and exemplary quotes (Table 1).

**Table 1:** Thematic analysis from two focus groups with people with opioid use disorder (OUD) (*n* = 9) at least 5 years post‐sustained virological response (SVR) through facilitated telemedicine for hepatitis C virus (HCV) care integrated into opioid treatment programmes (OTPs) in New York State.

Theme	Sub‐Theme		Example quote
A. Attitudes towards HCV and barriers to treatment among people with OUD	1. Feelings of shame, self‐blame and fear of disclosing HCV status		‘I didn't know what [other people] would think, or they'd drink after me’ [F2P5] ‘I was afraid to tell [romantic partner]’ [F1P3] ‘I couldn't blame nobody but myself’ [F1P2]
	2. Witnessing HCV‐related complications in a relative and its influence on HCV awareness		‘[My brother] got cancer of the liver’ [F1P2] ‘[My husband] was in the hospital, and he was told he had Hep C, because [their tattoo] got severely infected. Then, they found out I had it…. He also got liver cancer at the same time’ [F1P4] ‘My brother had [HCV]. He had cirrhosis of the liver’ [F2P5]
	3. Competing medical and social priorities		‘They [people with OUD] have got other things going on, more important things, and people are drug addicts. People are using [drugs]. They're not so inclined to get their health taken care of’ [F1P1] ‘At that point in time, with losing my daughter and splitting up with my husband, I didn't really care’ [F1P4]
	4. Perceiving HCV as a low‐risk medical comorbidity		‘I wasn't really sick from it, the Hep C didn't really affect me at all’ [F2P3] ‘Many people had it [HCV]. Everybody I've talked to either had it or have it. So, it wasn't really a big deal’ [F2P3] ‘Hepatitis C was on the lower scale of it, 'cause if you're Hep C [positive]…. You ain't got HIV’ [F1P2]
	5. Third‐party payer limitations restricting HCV treatment accessibility		‘First, they told me I had to get sober. I had to be sober for three months for them to even attempt the treatment’ [F2P2] ‘I went to the hospital to try to get a cure or try to get rid of it [HCV], and they told me they couldn't help me until I had cirrhosis of the liver and was almost dying, unless I wanted to pay $1500 for a pill or $35,000 for a cure’ [F2P5]
B. Embracing facilitated telemedicine for HCV care integrated into OTPs	1. The technical benefits of facilitated telemedicine	1. Convenience	‘It was easier than having to go to a different doctor and deal with more appointments’ [F1P3] ‘I thought it was quick and easy. Very easy. It was better than having to go over to the doctors’ [F2P5]
		2. Overcoming transportation barriers	‘I didn't have to go to another office and be with another [doctor], more waiting and more driving, and I live far away, so it's hassle to have to go somewhere else’ [F1P3] ‘I don't have transportation. I'm taking the bus, and I'm on a bike and a skateboard half the time, so that was great. Anything else would have been a hassle in my life’ [F1P1] ‘It was easier talking to the doctor over a computer than it was going to see him. I'd rather sit there, have him on my phone, and computer, doing it that way than having to go to see him, unless I had to get bloodwork or something… Way better than trying to find a ride or take the bus or waiting on these cabs’ [F2P3]
	2. The social value of care integrated into OTPs	1. Confidentiality and trust	‘[The OTP staff] didn't go around telling, “hey, everybody, he's got [HCV]”’ [F1P2] ‘I trusted everyone that was here, so not a problem’ [F1P4] ‘You trust the people here…. You're coming here because you trust them, and you want help. So, it's all hand‐in‐hand’ [F1P4]
		2. Empathetic facilitated telemedicine clinicians	‘[Telemedicine clinicians] were very kind. They listened. They talked to you like a person…. When I'd go to other doctors, I've got a lot [of negative things] to say about them, but the doctors here, the tele‐visit, they were great. I wouldn't change a thing…. It's a two‐way conversation. It's not like when you go to a regular doctor's visit. They look at you. It's like [the in‐person doctors] don't want to hear’ [F2P5] ‘First of all, I hate hospitals. Second of all, I dislike doctors. I really dislike doctors’ [F2P5] ‘[The telemedicine clinicians] were fine. They did their job. They took care of what was taken care of. They listened to what I had to say. And they were good doctors’ [F2P5]
		3. Patient‐centred care	‘[OTP staff] was great. She was easy to get along with. She made it easy to understand. She was down to earth. So, it was easy to talk to her as well’ [F2P5] ‘They gave me all the information I needed and assured me that it's not as bad as I thought it was and made me feel very comfortable’ [F2P2] ‘The information they were giving, sitting down and talking to you, and explaining everything to you’ [F1P4] ‘They listened to what I had to say’ [F2P5]
	3. The simplified modern HCV medication regimen	1. Short, well‐tolerated, and highly effective HCV medication	‘I think that doing the [interferon] treatment affected me more than Hep C’ [F2P3] ‘Are you kidding me? It was terrible…. Towards the end of the treatment, I was so wiped out…. The hair was falling out. It was terrible’ [F2P1] ‘It was a pill you take for 90 days’ [F1P1] ‘They said that it's one pill a day for 12 weeks. Not too many side effects’ [F2P3]
		2. One‐stop shop approach	‘It was much easier having given [HCV treatment] to us here with our [methadone] dose and having to get the HCV pill. It was much easier than having to remember to take it at home. It's a one‐stop shop’ [F1P4] ‘We're coming here every day anyways’ [F2P3] ‘You're already here, like [F2P3] said’ [F2P1] ‘I would say it was convenient, because I was here anyway’ [F1P2] ‘It worked out good, because I didn't have to go somewhere else. And you've got to come here anyways’ [F2P2]
C. Experiencing long‐term benefits from facilitated telemedicine across all aspects of HCV care and overall well‐being	1. Patients feeling grateful for receiving an HCV diagnosis		‘I was glad to know that I had found out’ [F1P1] ‘I was confident that everything was going to be better than it was, was glad, and felt safe that I was being taken care of. I had the right answers, and I knew basically exactly where it [HCV] was and how bad it was’ [F1P4]
	2. Patients feeling thankful for accessing HCV treatment that would have otherwise been inaccessible		‘I didn't have to get sober to do it [HCV treatment]’ [F2P2] ‘I was very lucky to get the treatment here’ [F2P5] ‘It [HCV treatment] was free. Medical things cost now. Everything costs, but especially medical help. So, knowing that it was free, and it was available at that time, was a good thing’ [F1P2] ‘You couldn't get no better than the way it went. That's all I can say. I couldn't ask for no better. Because I tried before, and it was a nightmare. From what the doctors were telling me, it's $1500 a pill, $35,000 for the cure. Got to wait till I'm dead, half dead before they even try to [treat you]. Then, I came here, and that was laid on me, and that was a Godsend. So, I was very lucky to get the treatment here. Then, [the research study] paid for it besides. It was a good thing’ [F2P5]
	3. Enhanced engagement in care through facilitated telemedicine		‘I just missed a dentist appointment because I fell asleep. I'd like to do more appointments on the phone or on video’ [F1P1] ‘I haven't [visited a hospital] since my daughter died, I haven't gone to a doctor…. If I had to go to the doctor [for HCV treatment], I probably wouldn't have gone. I have PTSD from being in there’ [F1P4]
	4. Enhanced HCV knowledge, awarenesspeer engagement in treatment		‘Now that I know a lot more about it, it's a little different…. You know you can't catch it by drinking after somebody, just through blood or blood contact’ [F2P5] ‘I get tested for it [HCV], just to make sure I didn't re‐contract it’ [F1P1] ‘I've learned a few things like to try not to re‐infect myself by using somebody else's needles or dirty needles’ [F2P5] ‘It [HCV] can progress to cirrhosis, you want to get treated, that you can infect others using razors or needles or saliva’ [F1P4] ‘I've told them what I did and how easy it was for me, but it's better if they jump on it and do it themselves’ [F1P1] ‘I think [friend's name] is stupid not to take the pill. It's a pill. You'll put anything else into your body, but you won't take something that's good for you’ [F1P4] ‘You just tell them about it, how easy it is, just a pill. You'd think people would jump on that, but people end up popping that pill’ [F1P4]
	5. Improved OUD recovery and whole health		‘I was using a lot of drugs before. Not using so much now…. I don't do needles no more’ [F2P5] ‘I was sober for a year or two after [SVR was achieved]’ [F1P1] ‘I definitely don't get high anymore’ [F1P2] ‘I don't use IV drugs anymore’ [F2P1] ‘When I got the [HCV] treatment done, I absolutely felt better, I had more energy…. My mental health was better…. My health feels a lot better…. I'm able to do more physical activity…. I feel more active and alive, and I'm happy that I got rid of it’ [F2P2] ‘I don't have to worry about it spreading to somebody else’ [F1P1] ‘The one thing I was able to do once I found out that I was better [cured] was hold my grandchildren a little closer’ [F2P5] ‘I don't have to worry about if I ever date again to tell that person I have it [HCV]’ [F1P3]

Abbreviations: HCV, hepatitis C virus; OTP, opioid treatment programme; OUD, opioid use disorder; PTSD, post‐traumatic stress disorder; SVR, sustained viral response.

### 3.1 Theme 1: Attitudes Towards HCV and Barriers to Treatment Among People With OUD

In the first theme, we describe participants' attitudes towards HCV before receiving HCV care through facilitated telemedicine. When asked about their initial feelings towards HCV, most described experiencing shame and fearing judgement from others upon disclosing their HCV status. For example, one participant shared: ‘*I didn't know what [other people] would think, or they'd drink after me [F2P5]’*. Another participant disclosed: ‘*I was afraid to tell [romantic partner] [F1P3]’*. Many participants also expressed a sense of personal responsibility for acquiring HCV, putting the ‘blame’ on themselves. One participant shared: ‘*I couldn't blame nobody but myself [F1P2]’*. Witnessing HCV‐related complications in a family member influenced participants' awareness of the disease, as many participants had relatives with cirrhosis and liver cancer. FGD participants acknowledged that the experiences of those acquaintances played a role in their decision to pursue HCV treatment.

Participants described encountering numerous barriers in their previous attempts to access HCV care. Many highlighted competing medical and social priorities that had interfered with seeking treatment, often placing other urgent concerns above their healthcare needs. The impact of OUD further complicated their efforts to engage in care, as described by a participant: ‘*They [people with OUD] have got other things going on, more important things, and people are drug addicts. People are using [drugs]. They're not so inclined to get their health taken care of [F1P1]’*. Another participant shared: ‘*At that point in time, with losing my daughter and splitting up with my husband, I didn't really care [F1P4]’*.

Another frequently cited barrier was the perception of HCV as a low‐risk medical condition, particularly due to its asymptomatic nature. For instance, one participant shared: ‘*I wasn't really sick from it, the Hep C didn't really affect me at all [F2P3]’*. Additionally, many participants compared HCV to HIV, often perceiving it as a lower‐priority condition. Another participant stated: ‘*Hepatitis C was on the lower scale of it, 'cause if you're Hep C [positive]…. You ain't got HIV [F1P2]*’, indicating a misconception that HCV–HIV coinfection is not possible, shaping participant's perception of risk. However, a minority of participants expressed significant fear and concern, equating HCV with HIV in terms of severity. One participant described: ‘*I felt like my life is over. I found out, and I felt like I was told that I had AIDS [F2P2]’*. Lastly, participants disclosed that third‐party payer restrictions posed significant challenges in accessing HCV treatment. One major barrier was the requirement for sobriety before becoming treatment‐eligible, as one participant shared: ‘*First, they told me I had to get sober. I had to be sober for 3 months for them to even attempt the treatment [F2P2]’*.

### 3.2 Theme 2: Embracing Facilitated Telemedicine for HCV Care Integrated Into OTPs

In the second theme, we summarise participants' experiences and views on receiving HCV care through facilitated telemedicine integrated into OTPs. This intervention encompassed three key aspects: (1) the technical aspect of telemedicine, (2) the social aspect largely attributed to integration of care within the OTP, and (3) hepatitis C medication dispensing with methadone. Accordingly, we organised Theme 2 into three sub‐themes that correspond to these aspects.

#### 3.2.1 Sub‐Theme 2.1 The Technical Benefits of Facilitated Telemedicine

Participants highly valued facilitated telemedicine for its convenience, describing it as a more comfortable way to access medical care than through referral to a conventional healthcare setting. A participant stated: ‘*It was easier than having to go to a different doctor and deal with more appointments [F1P3]’*. By utilising facilitated telemedicine, participants were able to overcome transportation barriers to access care. One participant shared: ‘*I don't have transportation. I'm taking the bus, and I'm on a bike and a skateboard half the time, so that was great. So, anything else would have been a hassle in my life [F1P1]’*. Another participant emphasised that for certain conditions where a ‘hands‐on’ patient interaction was not necessary, telemedicine was perceived as a more efficient option, stating:

It was easier talking to the doctor over a computer than it was going to see him. I'd rather sit there, have him on my phone, and computer, doing it that way than having to go to see him, unless I had to get bloodwork or something…. Way better than trying to find a ride or take the bus or waiting on these cabs.

[F2P3]

#### 3.2.2 Sub‐Theme 2.2 The Social Value of Care Integrated Into OTPs

Participants emphasised the social value of facilitated telemedicine and receiving care integrated into OTPs. Specifically, maintaining confidentiality in the OTP was particularly important. A participant stated: ‘*[The OTP staff] didn't go around telling, “hey, everybody, he's got [HCV]” [F1P2]’*. Trust in the OTP environment was consistently highlighted in the FGDs, with participants expressing, regarding the OTP staff, that they would ‘*trust everyone that was here’*. In addition, all participants reported positive experiences with the facilitated telemedicine clinicians, with many expressing a preference for facilitated telemedicine over in‐person visits. One participant explained their preference for the facilitated telemedicine clinicians due to their more empathetic approach:

[Telemedicine clinicians] were very kind. They listened. They talked to you like a person…. When I'd go to other doctors, I've got a lot [of negative things] to say about them, but the doctors here, the tele visit, they were great. I wouldn't change a thing…. It's a two‐way conversation. It's not like when you go to a regular doctor's visit. They look at you. It's like [the in‐person doctors] don't want to hear.

[F2P5]

Participants elaborated on their perceptions of the social value of care through the OTP as they described experiencing compassionate, patient‐centred care within the OTP. They shared how the OTP staff played a crucial role in facilitating appointment coordination and improving their understanding of the reasons for medical visits. One participant recalled an OTP staff member by name, emphasising their significant role in coordinating care: ‘*[OTP staff] was great. She was easy to get along with. She made it easy to understand. She was down to earth. So, it was easy to talk to her as well [F2P5]’*. Another participant highlighted the support they received from the OTP staff, stating, ‘*They gave me all the information I needed and assured me that it's not as bad as I thought it was and made me feel very comfortable [F2P2]’*. When asked about what participants found most helpful about the OTP staff, another participant emphasised their humane, patient‐centred approach to communication, stating, ‘*The information they were giving, sitting down and talking to you, and explaining everything to you [F1P4]’*.

#### 3.2.3 Sub‐Theme 2.3 The Simplified Modern HCV Medication Regimen

Many participants had either direct, personal experiences with interferon‐based HCV treatment or were familiar with it through relatives' and friends' experiences. Participants expressed frustration, often speaking angrily when recalling these experiences: ‘*I think that doing the treatment affected me more than Hep C [F2P3]’*. Another described: ‘*Are you kidding me? It was terrible…. Towards the end of the treatment, I was so wiped out…. The hair was falling out. It was terrible [F2P1]’*. In contrast, all participants agreed that modern HCV treatment with DAAs is easy, short, well‐tolerated, and highly effective. One participant recalled their experience receiving DAAs through facilitated telemedicine: ‘*They said that it's one pill a day for 12 weeks. Not too many side effects [F2P3]’*. Others also affirmed the simplicity of the treatment, describing it as ‘*a pill you take for 90 days [F1P1]’*.

Participants also appreciated the one‐stop shop approach when discussing their experiences receiving HCV care at the OTP. One participant shared: ‘*It was much easier having given [HCV treatment] to us here with our [methadone] dose and having to get the HCV pill. It was much easier than having to remember to take it at home. It's a one‐stop shop [F1P4]’*. Participants elaborated on this perception: ‘*We're coming here every day anyways [F2P3]’*.

### 3.3 Theme 3: Experiencing Long‐Term Benefits From Facilitated Telemedicine Across All Aspects of HCV Care and Overall Well‐Being

In the third theme, we identified participants' perspectives on an HCV cure that was achieved at least 5 years post‐SVR. Most participants expressed gratitude for having received an HCV diagnosis through the facilitated telemedicine trial. One participant stated: ‘*I was glad to know that I had found out [F1P1]’*, a sentiment with which others agreed. Many commented that without facilitated telemedicine, they would have never received HCV treatment. One participant noted: ‘*I didn't have to get sober to do it [F2P2]’*. Other participants highlighted the benefit of receiving treatment at no cost, while also expressing appreciation for the compensation provided for their participation in the study:

You couldn't get no better than the way it went. That's all I can say. I couldn't ask for no better. Because I tried before, and it was a nightmare. From what the doctors were telling me, it's $1500 a pill, $35,000 for the cure. Got to wait till I'm dead, half dead before they even try to [treat you]. Then, I came here, and that was laid on me, and that was a Godsend. So, I was very lucky to get the treatment here. Then, [the research study] paid for it besides. It was a good thing.

[F2P5]

Participants reported greater engagement in HCV care as a result of the facilitated telemedicine intervention. Many had previously avoided hospitals and medical appointments. One participant shared: ‘*I haven't [visited a hospital] since my daughter died, I haven't gone to a doctor…. If I had to go to the doctor [for HCV treatment], I probably wouldn't have gone. I have PTSD from being in there [F1P4]’*. Others felt that facilitated telemedicine made it easier to attend appointments, reducing the likelihood of missed visits: ‘*I just missed a dentist appointment because I fell asleep. I'd like to do more appointments on the phone or on video [F1P1]’*. Participants also reported increased awareness and knowledge of HCV, including its transmission modes, prevention strategies, and reinfection risk. One participant noted: ‘*Now that I know a lot more about it, it's a little different…. You know you can't catch it by drinking after somebody, just through blood or blood contact [F2P5]’*. Another participant reflected: ‘*I've learned a few things like to try not to re‐infect myself by using somebody else's needles or dirty needles [F2P5]’*. Other participants became more proactive about testing, as one participant described routine HCV screening post‐treatment: ‘*Just to make sure I didn't re‐contract it [F1P1]’*. Many participants had shared their knowledge with their peers, encouraging them to seek HCV treatment as well. One shared: ‘*I've told them what I did and how easy it was for me, but it's better if they jump on it and do it themselves [F1P1]’*. Another participant explained: ‘*I think [friend's name] is stupid not to take the pill. It's a pill. You'll put anything else into your body, but you won't take something that's good for you [F1P4]’*.

Participants described the impact of achieving an HCV cure as extending beyond the immediate health benefits related to viral clearance. Many participants shared that achieving an HCV cure also marked a turning point in their lives, helping them to maintain sustained sobriety. However, perceptions of sobriety varied among participants. For some, it meant abstaining from intravenous drug use solely, as one participant shared: ‘*I was using a lot of drugs before. Not using so much now…. I don't do needles no more [F2P5]’*. Others maintained sobriety for shorter periods: ‘*I was sober for a year or two after [SVR was achieved] [F1P1]’*. Many participants reported overall improvements in their whole health, energy levels, and mental health post‐SVR:

When I got the treatment done, I absolutely felt better, I had more energy…. My mental health was better…. My health feels a lot better…. I'm able to do more physical activity…. I feel more active and alive, and I'm happy that I got rid of it.

[F2P2]

Many participants felt relief after achieving an HCV cure, knowing they were no longer at risk of transmitting the virus to others: ‘*I don't have to worry about it spreading to somebody else [F1P1]’*. The sense of relief also helped strengthen personal relationships. For example, one participant shared that they felt more comfortable being around their grandchildren: ‘*The one thing I was able to do once I found out that I was better [cured] was hold my grandchildren a little closer [F2P5]’*.

## 4 Discussion

At least 5 years post‐SVR, people with OUD reported lasting benefits from receiving HCV care through facilitated telemedicine integrated into OTPs. These benefits included improved recovery from OUD and overall well‐being, highlighting the sustained impact of an HCV cure. Before receiving care through facilitated telemedicine, participants reported facing numerous barriers to accessing HCV treatment, such as competing medical and social priorities, perceiving HCV as a low‐risk medical comorbidity, and third‐party payer restrictions. Participants identified a triple benefit of the intervention across three key aspects. First, the telemedicine technical component offered convenience and reduced transportation barriers. Second, the social aspect of facilitated telemedicine integrated into OTPs created a trusted environment for people with OUD, ensured confidentiality, and fostered empathetic clinical care. Third, the simplicity of modern HCV medication, characterised by a favourable safety profile, short duration, and high effectiveness, was valued, particularly for the convenience of co‐dispensing HCV medications together with methadone in a one‐stop shop setting.

Most participants reported initially perceiving HCV as a low‐priority medical condition that did not require immediate medical attention. This perspective has commonly been reported in prior studies involving similar populations [26, 27, 28, 29]. Following HCV care, participants demonstrated improved understanding and increased awareness of HCV, including its transmission, prevention, and diagnosis. These results align with previous research by Muncan et al., which highlighted the role of SSPs in enhancing HCV knowledge when care is provided in an integrated format [30]. In our facilitated telemedicine trial, care integration occurred in OTPs, where participants experienced comparable benefits in knowledge improvement and care engagement [31]. Many participants continued to undergo regular HCV RNA assessment for reinfection even 5 years post‐SVR, indicating a shift in individual health‐seeking behaviours with a long‐lasting impact on broader public health efforts. While increased HCV knowledge is associated with a greater willingness to seek treatment, the quality of patient–provider interactions is also crucial to shape patients' perceptions and health decisions [32, 33].

People with OUD, who often experience both social exclusion and limited access to medical care, shared feelings of shame associated with a prior HCV diagnosis and manifestations of internalised stigma [34, 35, 36, 37]. Notably, participants identified OTPs as trusted, supportive environments that protected their privacy and offered non‐judgemental care, comparing them to traditional healthcare settings where participants were more concerned with unwanted disclosure of their HCV status. Their existing trust in the OTP setting and the case managers who facilitate the telemedicine encounters contributed to extending that trust to the facilitated telemedicine clinicians, promoting openness amongst participants to receiving HCV treatment through this intervention. In particular, case managers played a key role in understanding the competing priorities that hinder people with OUD from pursuing HCV care and in coordinating the delivery of personalised care [12].

Participants highly valued the empathy demonstrated by OTP staff and appreciated the compassionate approach of the facilitated telemedicine clinicians. In our previous investigation among a population of people with OUD in Greece who were largely inexperienced with telemedicine, participants expressed a preference for an initial in‐person appointment with their healthcare provider before transitioning to virtual care [16, 25]. In contrast, we did not observe similar preferences in this study. This difference underscores the way trust in virtual care may develop differently depending on the cultural context and speaks to the importance of patient‐centred telemedicine implementation [5, 25].

Beyond trust and empathy, participants emphasised the one‐stop shop nature of the intervention. By integrating medical care through facilitated telemedicine for HCV with OUD treatment within a setting that also provides essential social services, the intervention enabled the OTP to function as a ‘full‐service garage’, addressing a broad spectrum of healthcare issues within the same venue. Furthermore, interviews with OTP staff in a previous study indicated that facilitated telemedicine achieved full integration into OTP work flows [38]. While the literature largely supports the effectiveness of co‐locating HCV and OUD treatment in enhancing HCV care, widespread access to integrated medical and behavioural services remains limited [39, 40, 41]. A key finding of our study was that integrating HCV with OUD treatment within OTPs not only led to an HCV cure but also supported participants' recovery from OUD. Among participants who achieved an HCV cure, substance use declined significantly with minimal reinfections over 2 years follow‐up [15]. Six months post‐SVR, participants commented that, in many cases, achieving an SVR was their initial ‘win’ over a potentially lethal disease; an HCV cure increased self‐confidence and allowed for lifestyle changes [21]. Previous research has shown significant improvements in patient‐reported outcomes related to quality of life and work productivity during and shortly after treatment with DAAs [42, 43]. Here, we document that these positive changes in participants' overall health and OUD recovery persist for at least 5 years. These findings highlight the long‐term benefits of an HCV cure that extend beyond viral clearance and improved liver‐related outcomes. The improvement in OUD outcomes is likely expected based upon the interconnectedness between OUD and OUD‐related infectious diseases, namely HCV and HIV; treating one condition can positively influence the other. Vega et al., through in‐depth interviews with people with substance use disorder and HCV, found that participants believed addiction treatment should precede HCV treatment [44]. While OUD recovery might facilitate successful HCV treatment and vice versa, these processes are closely interconnected, providing a strong rationale for integrated OUD and HCV treatment [45].

An important limitation of this study is the small sample size. However, given the specific focus of the research question, we achieved data saturation [46]. Another limitation, inherent to the study design, is the reliance on participants' recollections of experiences from 5 years before the FGDs, which may be subject to recall bias [47]. Nonetheless, we have strong indications that our findings accurately reflect participants' experiences. This notion is supported by the consistency between these results and those from a prior qualitative assessment conducted 6 months post‐SVR with the same study population, where participants expressed similar perceptions [21]. In addition, our findings align with those reported in broader literature, which documents similar outcomes shortly after HCV treatment completion. A novel aspect of this study is that it confirms the long‐term benefits of an HCV cure. Finally, although our sample included a balanced male‐to‐female ratio, the majority of participants were White. Therefore, our findings may not be fully generalisable to other racial and ethnic groups, such as Black, Hispanic, Asian or migrant populations, where healthcare disparities, cultural issues, and socio‐economic factors may exert additional influences on long‐term outcomes following an HCV cure [6, 48, 49].

## 5 Conclusion

More than 5 years post‐SVR, people with OUD reported lasting benefits from an HCV cure that extended beyond viral clearance, including improvements in OUD recovery and overall health. Participants also expressed sustained satisfaction with the delivery of HCV care through facilitated telemedicine integrated within OTPs. This model effectively overcomes key obstacles previously encountered by this population, such as geographic challenges, stigma, limited provider empathy, distrust in traditional healthcare settings, and the high cost of treatment. These findings highlight the sustained impact of both an SVR and the care delivered through facilitated telemedicine. The long‐term benefits associated with facilitated telemedicine further support its viability as a sustainable public health intervention, well‐aligned with HCV elimination goals, and tailored to the needs of the high‐priority population of people with OUD.

## Author Contributions

**Zoi Papalamprakopoulou:** conceptualization, methodology, formal analysis, investigation, visualization, writing – original draft preparation, writing – review and editing. **Tin H. Dang:** conceptualization, methodology, formal analysis, writing – review and editing. **Christopher J. Gonzalez:** conceptualization, methodology, formal analysis, writing – review and editing. **Colin J. Tedesco:** investigation, data curation, writing – review and editing. **Paul F. Updike:** investigation, data curation, writing – review and editing. **Christina Corl:** investigation, data curation, writing – review and editing. **Arpan Dharia:** data curation, writing – review and editing. **Noelia Hernandez:** data curation. **Martin F. Shapiro:** conceptualization, methodology, funding acquisition, supervision, writing – review and editing. **Elaine Wethington:** conceptualization, methodology, formal analysis, supervision, writing – review and editing. **Andrew H. Talal:** conceptualization, methodology, formal analysis, funding acquisition, supervision, visualization, writing – review and editing.

## Disclosure

The statements in this study are solely the responsibility of the authors and do not necessarily represent the views of PCORI, its Board of Governors or Methodology Committee. Study funders were not involved in data collection, analysis or manuscript preparation.

## Ethics Statement

The study protocol was approved by the Biomedical Research Alliance of New York Institutional Review Board. The study adhered to the Helsinki Declaration principles.

## Consent

We obtained written informed consent from all study eligible participants before their participation and verbal consent before recording the FGDs.

## Conflicts of Interest

A.H.T. reported receiving non‐financial support from Abbott Laboratories and grants from Gilead Sciences, Novo Nordisk, AstraZeneca and Salix. A.H.T. has served as a committee/advisor for Gilead, AbbVie, Novo Nordisk and Madrigal. He also serves as Medical Director for Empath Medical Innovations Inc. The other authors declare no conflicts of interest.

## Supporting information

Additional File 1.

Additional file 2.

Additional file 3.

## Data Availability

The de‐identified focus group transcripts and analyses are available from the corresponding author upon reasonable request.
