# Supplementary material for: Long‐Term Benefits Following Hepatitis C Cure Through Facilitated Telemedicine; Experiences of People With Opioid use Disorder Five Years After Achieving a Sustained Virological Response
Source: Health Expect. 2025 Sep 24;28(5):e70404. doi: 10.1111/hex.70404 (PMC12457984; doi:10.1111/hex.70404)
Supplement: Supplementary file 1 — Additional File 1. [file HEX-28-e70404-s002.docx]

**Focus Group Interview Guide**

1. What did you know about hepatitis C before getting tested in the prior research study?

*Follow-up*: Do you know others who have been infected with hepatitis C?

2. How did you feel about your hepatitis C diagnosis when you first found out?

(Alternative question: what type of emotions did you feel when you first find out?)

*Follow-up*: How do you think hepatitis C has affected your health?

*Follow-up:* Do you think others who have found out about your hepatitis C diagnosis have treated you differently?

3. How was your experience with the clinic staff and doctors who told you about your hepatitis C diagnosis as part of the prior research study?

*Probe to encourage conversation:*

a. What did the clinic staff and doctors of the prior study do that was particularly helpful?

b. What did the clinic staff and doctors of the prior study do that was not as helpful?

c. Was there anything you wish your doctor did differently during that time?

4. When you were diagnosed with hepatitis C, what were you told about hepatitis C that you didn’t already know?

*Interviewer: let participants talk freely and use the following probes only if participants do not volunteer this information.*

*Probes to encourage conversation:*

a. What were you told about how you might treat hepatitis C?

b. What were you told about how hepatitis C might affect your health?

c. What were you told about how hepatitis C spreads to others?

*5.*What do you think about having received hepatitis C treatment coordinated at opioid treatment program?

Follow-up: What has been helpful for getting treatment at the opioid treatment program?

Follow-up: What has been challenging for getting treatment at the opioid treatment program?

6. What do you think about having received hepatitis C treatment through telemedicine?

Follow-up: What has been helpful for getting treatment through telemedicine?

Follow-up: What has been challenging for getting treatment through telemedicine?

Follow-up: Do you know anyone who has received hepatitis C treatment through telemedicine outside of the previous study?

7. How has your life changed since being cured of hepatitis C?

a. Have you been able to pursue any new activities? Any new accomplishments?

b. Have you been able to engage others into treatment for HCV?

8. Finally, is there anything else important that we have not discussed about diagnosis of HCV and the treatment?
