# Supplementary material for: Long‐Term Benefits Following Hepatitis C Cure Through Facilitated Telemedicine; Experiences of People With Opioid use Disorder Five Years After Achieving a Sustained Virological Response
Source: Health Expect. 2025 Sep 24;28(5):e70404. doi: 10.1111/hex.70404 (PMC12457984; doi:10.1111/hex.70404)
Supplement: Supplementary file 3 — Additional file 3. [file HEX-28-e70404-s003.docx]

**Additional file 3.** Consolidated criteria for reporting qualitative research (COREQ) Checklist.

The table below illustrates how and where (when applicable) our study addresses each item in the COREQ checklist.

| **Characteristic** | **Guide questions/description** | **Manuscript page number** | **Description or relevant text from manuscript.** |
| --- | --- | --- | --- |
| **Domain 1: Research team and reflexivity** | | | |
| *Personal Characteristics* | | | |
| 1. Interviewer/facilitator: | Which author/s conducted the interview or focus group? | Manuscript, page 11 | “The FGDs were led by an interviewer experienced in qualitative research (Z.P.)” |
| 2. Credentials | What were the researcher’s credentials? E.g. PhD, MD |  | Z.P.: MD  A.H.T: MD, MPH |
| 3. Occupation | What was their occupation at the time of the study? |  | **Z.P.** is a Research Support Specialist. She worked on patient recruitment, enrollment, focus group conduction, data analysis, and manuscript preparation.  **A.H.T.** is a tenured Professor of Medicine and Director of the Center for Clinical Care and Research in Liver Disease at the University at Buffalo Medical Center. He is a leading expert in liver disease. He conceptualized the study design and provided guidance and oversight throughout the entire project. |
| 4. Gender | Was the researcher male or female? |  | Z.P. is female and A.H.T is male. |
| 5. Experience and training | What experience or training did the researcher have? | Manuscript, pages 11-12 | “The FGDs were led by an interviewer experienced in qualitative research (Z.P.)”  “An expert in qualitative research design (E.W.) ensured the appropriateness of the interview guide for the target population, with input from all study team members.” |
| *Relationship with participants* | | | |
| 6. Relationship established | Was a relationship established prior to study commencement? | Page 10 | Yes. “Our study participants had previously received HCV treatment with DAAs through facilitated telemedicine between 2018 and 2020 as part of a multisite pragmatic trial conducted in 12 OTPs across New York State. Details on the study design are available elsewhere (ClinicalTrials.gov NCT02933970)” |
| 7. Participant knowledge of the interviewer | What did the participants know about the researcher? e.g. personal goals, reasons for doing the research- |  | Participants in the FGD were aware of the research's intent, either through communication with the facilitators or direct contact with the research study manager. Ultimately, participants were informed of the purpose of the investigation during the informed consent process. |
| 8. Interviewer characteristics | What characteristics were reported about the interviewer/facilitator? e.g. Bias, assumptions, reasons and interests in the research topic |  | **Z.P.** explained that she is an M.D. and has been working as the Research Support Specialist on the study. She elaborated that her research interests focus on exploring optimal healthcare strategies for people with opioid use disorder. |
| **Domain 2: Study design** | | | |
| *Theoretical framework* | | | |
| 9. Methodological orientation and Theory | What methodological orientation was stated to underpin the study? e.g. grounded theory,  discourse analysis, ethnography, phenomenology, content analysis | Manuscript, page 12-13 | “We analyzed the FGD transcripts using thematic analysis. The analysis team was comprised of four research members, referred to as “analysts”, (Z.P., T.H.D., C.J.G., A.H.T.), under the guidance of an expert in qualitative methodology (E.W.). The analysis followed an iterative process, beginning with independent analysis and coding of the FGD transcripts by each analyst following the completion of all FGDs.” |
| *Participant selection* | | | |
| 10. Sampling | How were participants selected? e.g. purposive, convenience, consecutive, snowball | Manuscript page 10 | “We employed purposive sampling to recruit participants from two OTPs in New York State. Inclusion criteria included individuals aged at least 18 years who had previously achieved SVR through facilitated telemedicine, remained active patients at the OTPs where they had received HCV treatment, and were able to provide written informed consent.” |
| 11. Method of approach | How were participants approached? e.g. face-to-face, telephone, mail, email | Manuscript, page 10 | “To facilitate recruitment, we employed study facilitators (C.C., C.T.) who were employed by the OTPs and are certified physician assistants. Initially, we identified a list of potential participants using a trial-specific database from the original trial period. Study facilitators then confirmed current patient status by cross-referencing this list with OTP electronic health records. Through a warm hand-off process mediated by the facilitators, a research team member (Z.P.) met with potential participants to assess their willingness to participate and confirm their eligibility. She explained all aspects of study involvement, obtained written informed consent, and led the FGDs.” |
| 12. Sample size | How many participants were in the study? | Manuscript, page 11 | “We conducted two FGDs in October 2024 and January 2025, with 4 and 5 participants, respectively (n = 9).” |
| 13. Non-participation | How many people refused to participate or dropped out? Reasons? | Manuscript, page 11 | “No participants prematurely discontinued the FGDs.” Of the 49 individuals previously enrolled in the facilitated telemedicine intervention at two OTPs, 44 remained active OTP patients at the time of follow-up. The remaining five had discontinued care due to loss of contact. All 44 eligible individuals were invited to participate in the FGDs, and all expressed interest. However, due to scheduling constraints and limited participant availability, we successfully coordinated two FGDs with a total of nine participants. |
| *Setting* | | | |
| 14. Setting of data collection | Where was the data collected? e.g. home, clinic, workplace | Manuscript, page 11 | “Each FGD lasted between 60 and 90 minutes and was held in a private room at the OTP.” |
| 15. Presence of non-participants | Was anyone else present besides the participants and researchers? | N/A | Only the participants and the researchers were present at the time of the FGD. |
| 16. Description of sample | What are the important characteristics of the sample? e.g. demographic data, date. | Manuscript, page 13 | “Participants had a mean age of 52.6 years (standard deviation = 13.7), 66.6% (6/9) were male, and 88.8% (8/9) identified as White.” |
| *Data collection* | | | |
| 17. Interview guide | Were questions, prompts, guides provided by the authors? Was it pilot tested? | Manuscript, page 11-12  Additional file 1 | “We developed a semi-structured interview guide featuring open-ended, non-stigmatizing, and non-leading questions to facilitate discussions about prior experiences with HCV treatment and the facilitated telemedicine intervention. Example questions included: *“What did you know about hepatitis C before getting tested in the prior research study?”*, *“What do you think about having received hepatitis C treatment through telemedicine?”*, and “*How has your life changed since being cured of hepatitis C?”* The complete interview guide is included in Additional File 1. To encourage more in-depth discussions, we used probes to further explore participants’ perspectives and experiences.”  An additional file contains the complete interview guide [see Additional file 1]. |
| 18. Repeat interviews | Were repeat interviews carried out? If yes, how many? | N/A | No repeat interviews were performed. |
| 19. Audio/visual recording | Did the research use audio or visual recording to collect the data? | Manuscript, page 12 | “The FGDs were audio-recorded and professionally transcribed by the qualified external language service provider Ubiqus/Acolad.” |
| 20. Field notes | Were field notes made during and/or after the interview or focus group? | N/A | Field notes were not made during the FGD. |
| 21. Duration | What was the duration of the interviews or focus group? | Manuscript, page 11 | “Each FGD lasted between 60 and 90 minutes…” |
| 22. Data saturation | Was data saturation discussed? | Manuscript, page 13 | “We determined that thematic saturation was reached when no new themes or insights emerged, even with the potential inclusion of additional FGDs.” |
| 23. Transcripts returned | Were transcripts returned to participants for comment and/or correction? | N/A | The transcripts were not returned to the participants for comment and/ or correction. |
| **Domain 3: analysis and findings** | | | |
| *Data analysis* | | | |
| 24. Number of data coders | How many data coders coded the data? | Manuscript, page 12-13 | “The analysis team was comprised of four research members, referred to as “analysts”, (Z.P., T.H.D., C.J.G., A.H.T.), under the guidance of an expert in qualitative methodology (E.W.).” |
| 25. Description of the coding tree | Did authors provide a description of the coding tree? | Manuscript, page 12-13  Additional File 2 | “The analysis followed an iterative process, beginning with independent analysis and coding of the FGD transcripts by each analyst following the completion of all FGDs. At this stage, analysts coded the transcripts, identified subthemes, consolidated the themes into preliminary higher-level themes, and selected representative quotations. The team then held a series of weekly meetings to discuss the preliminary findings, refine themes, and resolve disagreements. When disagreements arose, analysts revisited the original recordings and transcripts until consensus was reached. In the final stage, all team members provided input on the findings. At the conclusion of the process, all study team members agreed on the findings and produced a comprehensive codebook that is included in Additional File 2.” |
| 26. Derivation of themes | Were themes identified in advance or derived from the data? | Manuscript, page 12-13 | The themes were derived from the data.  “At this stage, analysts coded the transcripts, identified subthemes, consolidated the themes into preliminary higher-level themes, and selected representative quotations.” |
| 27. Software | What software, if applicable, was used to manage the data? | N/A |  |
| 28. Participant checking | Did participants provide feedback on the findings? | N/A | The participants of the FGDs did not provide feedback on the findings. Nevertheless, we have shared initial findings of the analysis with the study facilitators. Both the facilitators and the analysis team were individuals who had extensive experience working with the opioid use disorder patient population. All members of the analysis team agree with the findings as outlined in the submitted manuscript. |
| *Reporting* | | | |
| 29. Quotations presented | Were participant quotations presented to illustrate the themes / findings? Was each quotation identified? e.g. participant number | Manuscript, pages 13-21, Figure | Yes, representative participant quotations are presented to illustrate the findings and are labeled with a unique participant number to protect participant anonymity. |
| 30. Data and findings consistent | Was there consistency between the data presented and the findings? | Manuscript, pages 13-21,  Figure | Yes, the themes serve as evidence of the study findings. |
| 31. Clarity of major themes | Were major themes clearly presented in the findings? | Manuscript, pages 13-21,  Figure | Yes, the major themes are clearly presented in the results section, accompanied by example participant quotations.  “We identified three FGD themes each corresponding to pre-, during, or post-intervention of facilitated telemedicine: (1) attitudes toward HCV and barriers to treatment among people with OUD, (2) embracing facilitated telemedicine for HCV care integrated into OTPs, and (3) experiencing long-term benefits from facilitated telemedicine across all aspects of HCV care and overall well-being. The figure illustrates the key FGD themes, subthemes, and exemplary quotes.” |
| 32. Clarity of minor themes | Is there a description of diverse cases or discussion of minor themes? | Manuscript, pages 13-21, | Yes, we have described diverse cases and minor themes throughout the results section. |

Tong A, Sainsbury P, Craig J. Consolidated criteria for reporting qualitative research (COREQ): a 32-item checklist for interviews and focus groups. International Journal for Quality in Health Care. 2007;19(6):349-57
